# Scorpions and Their Human Mortality Report in Iran: A Review Article

**Published:** 2019-12

**Authors:** Faranak FIROOZFAR, Abedin SAGHAFIPOUR, Nahid JESRI

**Affiliations:** 1. Vector-Borne Diseases Research Center, North Khorasan University of Medical Sciences, Bojnurd, Iran; 2. Department of Public Health, Faculty of Health, Qom University of Medical Sciences, Qom, Iran; 3. Remote Sensing & GIS Center, Shahid Beheshti University, Tehran, Iran

**Keywords:** Scorpions, Mortality, Spacial distribution, Iran

## Abstract

**Background:**

The scorpions have enjoyed medical importance from ancient times because of their morphological structure and venom. The identification of the species of these arthropods has been more emphasized than any other aspects. The purpose of this study was to determine scorpion’s fauna and mortality rates of their victims as reported in Iran.

**Methods:**

In this review, published documents during 1966–2018 related to medically important scorpions and mortality reports due to scorpionism were searched in the data bases such as Web of Science, PubMed, Scopus, Google Scholar and etc., using key words including scorpion, species, classification, Iran, family, species, and the names of all 31 provinces of Iran.

**Results:**

Overall, 169 sources were found. Based on the STROBE checklist, the quality of the documents was also considered, and ultimately, 95 sources were selected. Sixty-four species of scorpions have been identified in Iran and 86% of the species belong to the Buthidae family, and the rest (9.5%, 4.5%) belong to the Hemiscorpiidae and Scorpionidae families, respectively. These species live mostly in tropical regions of Iran. The dangerous scorpions and their mortality reports are often recorded from southern regions such as Khuzestan and Hormozgan provinces.

**Conclusion:**

Due to the significant increase in the abundance of scorpions in Iran over the past 30 years, it is expected that this trend will continue by researchers, and the fauna of scorpions is regularly reconsidered.

## Introduction

Scorpions have been living on the earth for more than 450 million years. These creatures belong to the class: Arachnida; Lamarck, 1801. They have always been considered by humans because their painful and deadly stings. Scorpions are nocturnal arthropods. They have venom stings that are used to feed upon insects and self-defense (1,2). Their breeding places are mostly in the desert and in non-residential areas, but if their nests are destroyed, they also enter human dwellings (3).

Scorpions are hazardous to humans as they have toxic and deadly stings. The highest human mortality rate due to poisonous arthropods in the world is related to scorpionism (4, 5).

The scorpions are distributed in the world between the latitudes 23°N and 38°S of the equator (6, 7). In 1997, Kovarik categorized Iran's scorpions into three families: Buthidae, Scorpionidae, and Diplocentridae (8). Other researchers have mentioned four families of scorpions in Iran: Buthidae, Scorpionidae, Hemiscorpiidae, and Diplocentridae (9). Nevertheless, in 2016, Dehghani et al. reported three families for this anthropode in Iran: Buthidae, Hemiscorpiidae and Scorpionidae (10).

The scorpion sting (scorpionism) is considered as one of the health problems in tropical and sub-tropical countries, such as Iran (11–14). Due to the diversity of scorpion species in the vast zone of Iran, from the southern islands in the Persian Gulf to the northernmost regions, scorpion stings (scorpionism) have always been considered as one of the medical issues in most areas of Iran (10,15). Many victims of these venomous arachnids have been reported from different regions of Iran (16). According to statistics released by CDC of the Iranian Ministry of Health and Medical Education, Iran has the second rank of venomous animal bites in the world by a recording of about 250,000 bites, following Mexico (17). In addition, many deaths caused by scorpions occur in Iran, especially in southern and southern tropical regions of the country (18). For instance, scorpionism incidence rates and mortality of scorpionism were estimated to be 54.8 to 66/100000 populations in Iran during 2002–2011, respectively (13). Furthermore, the mortality of scorpion stings in Iran is about 10 times greater than snakebites as one of the health problem related to other Iranian dangerous animals and the human deaths associated to scorpion stings are about one third to one second of the snakebites (19, 20).

It seems that having enough information on medically important scorpions, their geographical distribution in different regions of the country, and the mortality reports of scorpionism can help health policy makers plan for the prevention of scorpion sting cases and design effective interventions to reduce scorpion sting cases.

Therefore, we aimed to determine the medically important scorpions and their mortality report in Iran, using Geographical Information System.

## Materials and Methods

### Study area

Iran is located in southwest Asia and the Middle East with an area of 1.648 million km^2^ and a population of 79, 926, 270, based on the last census in 2016. This country is divided geographically into 31 provinces. Iran has a high climatic diversity. From the north to the south, it has different climate zones. The four climates of Iran are as follows: 1) the moderate and humid climate (northern Iran), 2) the warm and dry climate (considerable parts of central regions), 3) the cold and mountainous climate (west and northwest of Iran) and 4) the warm and humid climate (southwest towards southwest of Iran) (21).

### Study design

**Fig. 1:**
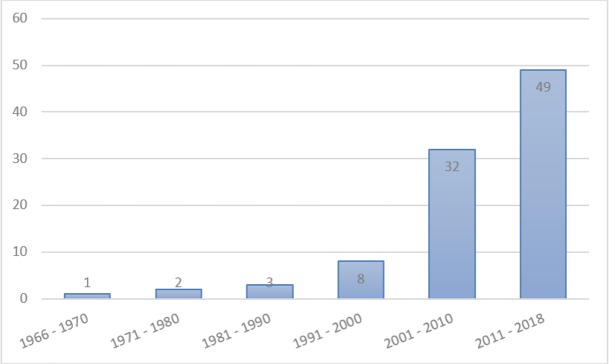
Historical trend of published documents in relation with scorpions, scorpionism and their reported mortality in Iran.

The present study is a review on medically important scorpions, as well as their mortality reports from all 31 provinces of Iran. The results of this study were obtained by searching related documents in scientific databases including Web of Science, PubMed, Medline, Scopus, Systematic Review, SID, Iran Medex, and Google Scholar. The manuscripts related to the present study were searched and extracted by two researchers independently using MeSH keywords such as Scorpion, Classification, Iran, Identification, Study, Mortality, Family and Species, from 1966 to 2018 (Fig. 1). Moreover, MSc theses and Ph.D. dissertation for those students who graduated in Medical Entomology, Vector Control, and Medical Entomology were reviewed from the libraries of medical universities of Iran, such as Tehran, Shiraz, Ahvaz, Urmia, Bandar Abbas, Hamadan, Tarbiat Modares, and others. Data categorization was performed by searching articles related to Provinces, Scorpions, Medicaly important scorpions, and mortality reports of scorpionism from different parts of Iran.

Inclusion criteria were as follows: documents that mentioned scorpion fauna or indicated on mortality of scorpion stings from each of the 31 provinces of Iran; identification of scorpions based on valid keys, mortality reports from the Center for Disease Control of the Ministry of Health in the form of an epidemiological survey forms that approved by the General physicians. In addition, the case reports on the scorpion deaths published in Persian or English journals were considered. Articles and theses that were not related to the subject of this study and faunistic studies of scorpions with incomplete sample size or invalid identification keys were excluded from the study.

At this stage, the Strengthening the Reporting Observational Studies in Epidemiology (STROBE); the standard STROBE checklist, which has 22 items, was used to assess the quality of the documents. This checklist evaluates all aspects of methodology including the study design, sampling methods, measurement of variables, data analysis, and so on (22). The minimum score for entering articles in the study was considered to be 15 .5 points. The studies with scores less than 15.5 were excluded.

High and medium-quality documents, which received minimum 16 points out of 44 points in the checklist, were included in the present study. Thus, the data collected from these documents were entered into Microsoft Office Excel 2010 for 12 dangerous and medically important scorpions of Iran. ArcGIS software ver. 9.3 (http://www.esri.com/arcgis) was employed to provide the special distribution maps of scorpions and their mortality reports.

## Results

Overall, 169 potentially related documents were found. According to the inclusion and exclusion criteria and the qualitative evaluation of documents, 74 sources were eliminated. Ultimately, 95 related sources were extracted and included in the study (Fig. 1). Iranian scorpions can be classified as three families: Buthidae, Scorpionidae, and Hemiscorpiidae, including 64 species and 20 genera. Of these scorpions, 55 species (86%) belong to the Buthidae family, six (9.5%) and three (4.5%) species were reported to belong to the Hemiscorpiidae and Scorpionidae families, respectively.

**Fig. 2:**
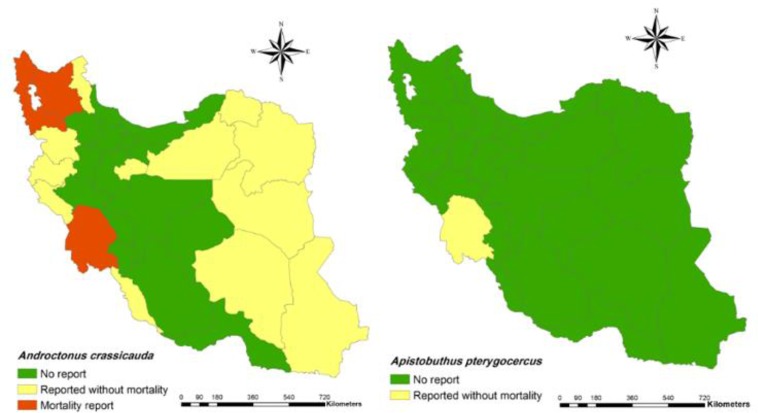
Presence of *Androctonus crassicauda* (left) and *Apistobuthus pterygocercus* (right) and their mortality report in Iran at the county

**Fig. 3:**
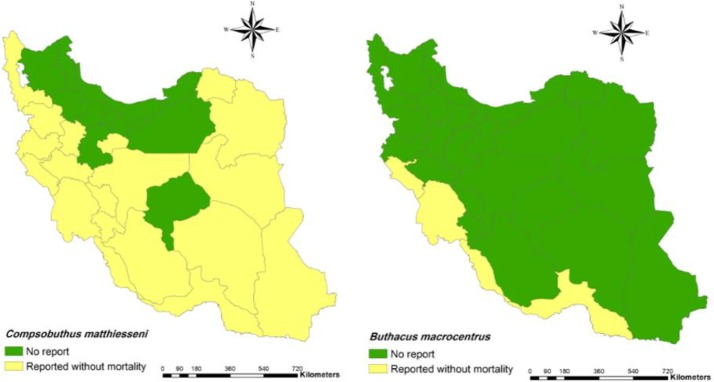
Presence of *Compsobuthus matthiesseni* (left) and *Buthacus macrocentrus* (right) and their mortality report in Iran at the county

**Fig. 4:**
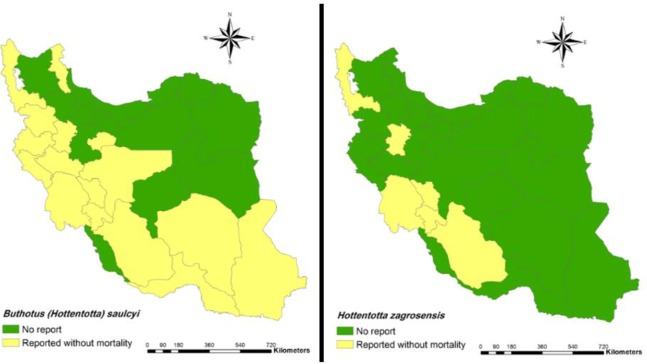
Presence of *Buthotus (Hottentotta) saulcyi* (left) and *Hottentotta zagrosensis* (right) and their mortality report in Iran at the county

**Fig. 5:**
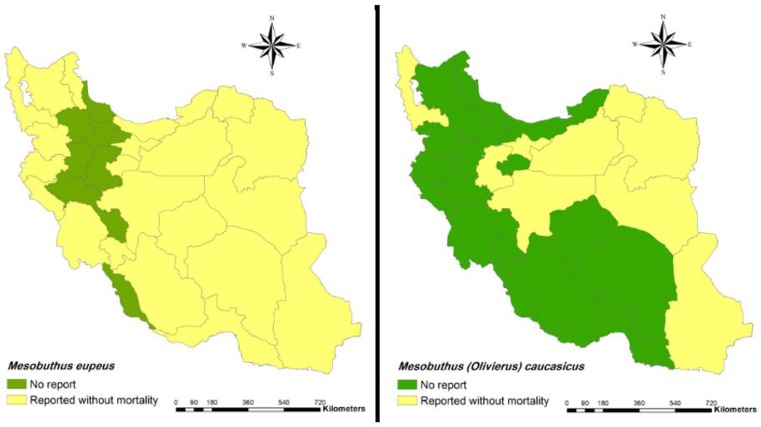
Presence of *Mesobuthus eupeus* (left) and *Mesobuthus caucasicus* (right) and their mortality report in Iran at the county

**Fig. 6:**
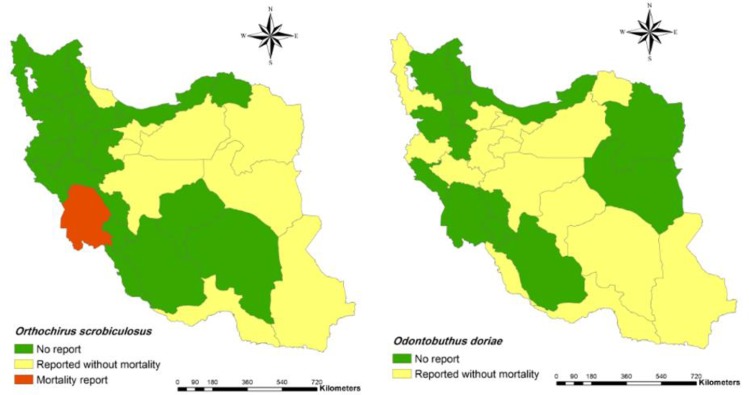
Presence of *Orthochirus scrobiculosus* (left) and *Odontobuthus doriae* (right) and their mortality report in Iran at the county

- Family Buthidae (C. L. Koch, 1837)
  1.1 Genus *Androctonus*
    1. *Androctonus crassicauda* (Olivier, 1807) (Note 1, Fig.2)
      **Distribution**: Bushehr, Semnan, Khuzestan, Ilam, Kurdistan, Razavi Khorasan, South Khorasan, Kermanshah, Kerman, Sistan and Baluchestan, Qom, West Azerbaijan, East Azerbaijan and Ardabil (7, 9, 12, 23–31).
    2. *Androctonus baluchicus* (Lournco 2005)
      **Distribution**: Sistan and Baluchestan (9, 24).
    3. *Androctonus robustus* (Kovařík & Ahmed, 2013)
      **Distribution**: Sistan and Baluchestan (24).
1.2 Genus *Apistobuthus*
    4. *Apistobuthus pterygocercus* (Finnegan, 1932) (Fig.2)
      **Distribution**: Khuzestan (24).
    5. *Apistobuthus susanae* (Lourenço, 1998)
      **Distribution**: Khuzestan, Lorestan (9, 32–34).
1.3 Genus *Buthacus*
    6. *Buthacus leptochelys* (Hemprich & Ehrehnberg, 1829)
      **Distribution**: Khuzestan, Bushehr and Hormozgan (24).
    7. *Buthacus macrocentrus* (Ehrenberg, 1828) (Fig.3)
      **Distribution**: Bushehr, Khuzestan, Hormozgan and Ilam ( 7, 9, 31, 33, 35).
    8. *Buthacus tadmorensis* (Simon, 1829)
      **Distribution**: Ilam (24).
1.4 Genus *Compsobuthus*
    9. *Compsobuthus garyi* (Lourenço et al Vachon, 2001)
      **Distribution**: Khuzestan (9, 33, 36).
    10. *Compsobuthus jakesi* (Kovařík, 2003)
      **Distribution**: Bushehr, Khuzestan, and Ilam (9 , 23, 33, 37, 38).
    11. *Compsobuthus kafkai* (Kovařík, 2003)
      **Distribution**: Sistan and Baluchestan (37).
    12. *Compsobuthus kaftani* (Kovařík, 2003)
      **Distribution**: Yazd, Isfahan and Kerman (9 , 37, 39– 41).
    13. *Compsobuthus matthiesseni* (Birula, 1905) (Fig.3)
      **Distribution**: Bushehr, Chaharmahal and Bakhtiari, Fars, Hamedan, Kerman, Kohgiluyeh and Boyer-Ahmad, Kurdistan, Lorestan, Markazi, Qom, Khuzestan, Hormozgan, Khorasan, Kermanshah, Ilam, Kurdistan, West Azarbaijan, Isfahan and Sistan and Baluchestan (4, 9, 23, 24, 27, 29, 33, 37, 38, 42–50).
    14. *Compsobuthus persicus* (Navidpour et al, 2008)
      **Distribution**: Fars, Bushehr (9, 23, 51).
    15. *Compsobuthus petriolii* (Vignoli, 2005)
      **Distribution**: Fars (9, 52).
    16. *Compsobuthus plutenkoi* (Kovařík, 2003)
      **Distribution**: Hormozgan (9, 37).
    17. *Compsobuthus acutecarinatus* (Simon, 1882)
      **Distribution**: Fars, Bushehr (24).
    18. *Compsobuthus rugosulus* (Pocock, 1900)
      **Distribution**: Fars, Bushehr (37, 46, 53).
    19. *Compsobuthus sobotniki* (Kovařík, 2003)
      **Distribution**: Hormozgan (9, 37).
1.5 Genus *Hottentotta*
    20. *Hottentotta zagrosensis* (Kova řík, 2003) (Fig.4)
      **Distribution**: Chaharmahal and Bakhtiari, Khuzestan, Fars, Kohgiluyeh and Boyer-Ahmad, Lorestan and West Azarbaijan (9, 24, 33, 34, 44, 54, 55).
    21. *Hottentotta schach* (Birula, 1905)
      **Distribution**: Fars, Khuzestan (9, 31, 33, 42).
    22. *Hottentotta saulcyi* (Simon, 1880) (Fig.4)
      **Distribution**: Lorestan, Hamedan, Chaharmahal and Bakhtiari, Khuzestan, West Azarbaijan, Kermanshah, Hormozgan, Ilam, Sistan and Baluchestan, Kurdistan, Kohgiluyeh and Boyer-Ahmad, Fars, Isfahan, Kerman and Ardabil, Qom (4, 7, 8, 12, 24, 29, 31, 33, 44, 45, 49, 56–62).
    23. *Hottentotta lorestanus* (Navidpour et al, 2010)
      **Distribution**: Lorestan (34).
    24. *Hottentotta khoozestanus* (Navidpour et al, 2008)
      **Distribution**: Khuzestan (63).
    25. *Hottentotta jayakari* (Pocock, 1895)
      **Distribution**: Qom, Hormozgan, Fars and Sistan and Baluchestan (4, 12, 24).
    26. *Hottentotta alticola* (Pocock, 1895)
      **Distribution**: Khuzestan, Lorestan, Hormozgan, Sistan and Baluchestan, Kermanshah (24).
1.6 Genus *Iranobuthus*
    27. *Iranobuthus krali* (Kova řík, 1997)
      **Distribution**: Fars and Isfahan (8, 39, 51).
1.7 Genus *Kraepelinia*
    28. *Kraepelinia palpator* (Birula, 1903)
      **Distribution**: Kerman and Yazd (40, 42, 57, 64).
1.8 Genus *Liobuthus*
    29. *Liobuthus kessleri* (Birula, 1898)
      **Distribution**: Razavi Khorasan (57).
1.9 Genus *Mesobuthus*
    30. *Mesobuthus eupeus* (C. L. Koch, 1839) (Fig.5)
      **Distribution**: Ardabil, Kerman, Isfahan, Markazi, Mazandaran, Sistan and Baluchestan, Yazd, Kohgiluyeh and Boyer-Ahmad, Semnan, Fars, Khuzestan, Hormozgan, Golestan, Tehran, Kurdistan, Kermanshah, Ilam, West Azarbaijan, Razavi Khorasan, South Khorasan, West Azerbaijan, East Azerbaijan, Ardabil, Qom (4, 7, 8, 9, 12, 24, 26, 27, 28, 29, 31, 39, 40, 41, 47, 60, 61, 62, 65–71).
    31. *Mesobuthus macmahoni* (Navidpour et al 2011)
      **Distribution**: Sistan and Baluchestan, Kerman (24, 41).
    32. *Mesobuthus phillipsii* (Mirhashemi et al 2011)
      **Distribution**: Khuzestan, Hormozgan (33, 72, 73).
    33. *Mesobuthus zarudnyi* (Birula, 1900)
      **Distribution**: Khuzestan, Hormozgan (24).
    34. *Mesobuthus vesiculatus* (Pocock, 1899)
      **Distribution**: Tehran, Isfahan, Yazd (39, 40, 74).
    35. *Mesobuthus caucasicus* (Nordmann, 1840) (Fig.6)
      **Distribution**: Sistan and Baluchestan, Isfahan, Khorasan, Tehran, Markazi, Semnan, West Azerbaijan, East Azerbaijan, Ardabil (27, 39, 42, 53, 57, 60, 61, 65, 71).
1.10 Genus *Odontobuthus*
    36. *Odontobuthus bidentatus* (Lourenço et Pézier, 2002)
      **Distribution**: Lorestan, Hormozgan and Khuzestan (24, 75).
    37. *Odontobuthus doriae* (Thorell, 1876) (Fig.6)
      **Distribution**: Hormozgan, Khuzestan, Razavi Khorasan, Kerman, Yazd, Isfahan, Markazi, Qazvin, Tehran, Alborz, Semnan, West Azarbaijan, Kermanshah, Bushehr, Hamedan, Hormozgan, Sistan and Baluchestan, Qom (4, 8, 12, 24, 28, 29, 31, 68, 70, 76–82).
    38. *Odontobuthus odonturus* (Pocock, 1897)
      **Distribution**: Khuzestan, Fars, Bushehr, Kermanshah, Ilam, Yazd, Sistan and Baluchestan, Qom (12, 24, 29, 46, 68).
    39. *Odontobuthus tavighiae* (Navidpour et al. 2013)
      **Distribution**: Hormozgan (24, 77).
    40. *Odontobuthus tirgari* (Mirhashemi et al 2012)
      **Distribution**: South Khorasan and Razavi Khorasan (24).
1.11 Genus *Orthochirus*
    41. *Orthochirus farzanpayi* (Vachon et Farzanpay, 1987)
      **Distribution**: Bushehr, Kerman, Hormozgan, Khuzestan, South Khorasan (4, 23, 24, 33 , 41, 57, 70, 83).
    42. *Orthochirus fuscipes* (Pocock, 1900)
      **Distribution**: Sistan and Baluchestan (53, 84).
    43. *Orthochirus gruberi* (Kovařík et al 2006) Distribution: Kerman (41, 83).
    44. *Orthochirus iranus* (Kovařík, 2004)
      **Distribution**: Lorestan, Khuzestan, Ilam, Kohgiluyeh and Boyer-Ahmad, Hamedan and Bushehr (7, 23, 33, 38, 44, 48, 62, 84).
    45. *Orthochirus scrobiculosus* (Birula, 1900) (Note 2, Fig.6)
      **Distribution**: Khuzestan, Hormozgan, Tehran, Sistan and Baluchestan, Qom, Isfahan, Razavi Khorasan, South Khorasan, Gilan, Semnan, Kermanshah, Ilam (4, 8, 9, 12, 24, 27, 29, 49, 56, 64, 65, 71, 84).
    46. *Orthochirus stockwelli* (Lourenço et Vachon, 1995)
      **Distribution**: Khuzestan, Ilam, Hormozgan and Bushehr (23, 33, 83, 85).
    47. *Orthochirus varius* (Kovařík, 2004)
      **Distribution**: Hormozgan (24).
    48. *Orthochirus zagrosensis* (Kovařík, 2004)
      **Distribution**: Khuzestan, Kohgiluyeh and Boyer-Ahmad, Isfahan, Yazd and Kerman (40, 41, 44, 84).
1.12 Genus *Anomalobuthus*
    49. *Anomalobuthus talebii* (Teruel et al 2014)
      **Distribution**: South Khorasan (24, 86).
1.13 Genus *Polisius*
    50. *Polisius persicus* (Fet, Capes et Sissom, 2001)
      **Distribution**: Ilam, Sistan and Baluchestan, Isfahan and Kerman (7, 38, 39, 41, 87).
1.14 Genus *Razianus*
    51. *Razianus zarudnyi* (Birula, 1903) **Distribution**: Chaharmahal and Bakhtiari, Sistan and Baluchestan, Lorestan, Khuzestan, Hormozgan, Ilam, Kohgiluyeh and Boyer-Ahmad (7, 33, 34, 38, 42, 44, 45, 47, 57, 62).
1.15 Genus *Sassanidotus*
    52. *Sassanidotus gracilis* (Birula, 1900)
      **Distribution**: Sistan and Baluchestan, Hormozgan, Kerman (24, 41, 42, 83).
    53. *Sassanidotus zarudnyi* (Birula, 1903)
      **Distribution**: Sistan and Baluchestan, Hormozgan, Tehran (24, 42, 57, 83).
1.16 Genus *Vachoniolus*
    54. *Vachoniolus iranus* (Navidpour et al., 2008)
      **Distribution**: Khuzestan (33).

II. Family Scorpionidae
2.1 Genus *Scorpio*
    55. *Scorpio maurus townsendi* (Pocock, 1900)
      **Distribution**: Khuzestan, Isfahan, Khorasan, Chaharmahal and Bakhtiari, Lorestan, Kohgiluyeh and Boyer-Ahmad, Kurdistan, Gilan, Fars, West Azarbaijan, Qazvin, Alborz, Semnan, Bushehr, Kermanshah, Ilam, Sistan and Baluchestan and Ardabil (9, 12, 23, 24, 31, 33, 38, 39, 44, 45, 46, 48, 59, 60, 62, 81).
  2.2 Genus *Nebo*
    56. *Nebo henjamicus* (Franck, 1980)
      **Distribution**: Hormozgan (24).
    57. *Nebo n. sp* (Dehghani 2008)
      **Distribution**: Kerman (28).

**Fig. 7:**
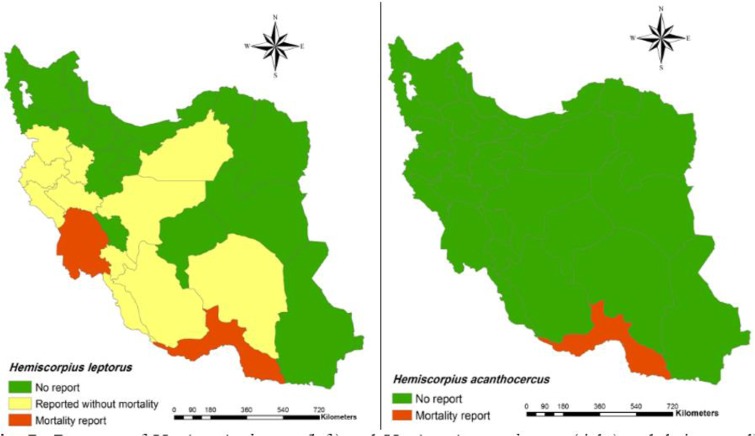
Presence of *Hemiscorpius lepturus* (left) and *Hemiscorpius acanthocercus* (right) and their mortality report in Iran at the county

III. Family Hemiscorpiidae
1.3 Genus *Hemiscorpius*
58. *Hemiscorpius acanthocercus* (Monod et Lourenço, 2005) (Note 3, Fig. 7)
      **Distribution**: Hormozgan (24, 88).
    59. *Hemiscorpius enischnochela* (Monod et Lourenço, 2005)
      **Distribution**: Hormozgan, Khuzestan (24, 88).
    60. *Hemiscorpius gaillardi* (Vachon, 1974)
      **Distribution**: Sistan and Baluchestan, Kerman (27, 60, 88).
    61. *Hemiscorpius lepturus* (Peters, 1862) (Note 4, Fig.7)
      **Distribution**: Khuzestan, Semnan, Fars, Kurdistan, Hormozgan, Bushehr, Ilam, Lorestan, Kermanshah, Isfahan, Hamedan, Kohgiluyeh and Boyer-Ahmad and Kerman (4, 7 , 23–25, 28, 31, 33, 38, 41, 44– 49, 58, 59, 62, 70, 72, 88, 89).
    62. *Hemiscorpius persicus* (Birula, 1903)
      **Distribution**: Sistan and Baluchestan (42, 88).
    63. *Hemiscorpius kashkayi* (Karataş and Gharkheloo 2013)
      **Distribution**: Khuzestan (24, 90).

In addition, spatial distribution of medicaly important scorpions and their mortality report in Iran has presented with geographical maps (Fig 2–7).

64. *Hemiscorpius shahii*
  **Distribution**: Hormozgan (91).
  **Note 1:** *Androctonus crassicauda* is known as the medically important scorpion species in the Middle East (92). Mortality report due to *Androctonus crassicauda* stings have been recorded in Khuzestan, North West of Iran, West Azerbaijan, and East Azerbaijan (60, 93, 94).
  **Note 2:** *Orthochirus scrobiculosus:* Mortality report of *Orthochirus scrobiculosus* has been recorded only in Khuzestan province, southwest of Iran (5).
  **Note 3:** The death report of *Hemiscorpius acanthocercus* has been recorded in Hormozgan province, south of Iran (95).
  **Note 4:** *Hemiscorpius lepturus:* The death of this scorpion species has been reported in Khuzestan and Hormozgan provinces; southwest and south of Iran (96, 97).

## Discussion

Of the three scorpion families reported in Iran (Buthidae, Scorpionidae, and Hemiscorpiidae), the family Buthidae has the highest frequency (86%). The classification of species, genera, families, and superfamilies of reported scorpions in Iran has been expanded and modified in a relatively short period. In addition, considering the use of new identification techniques in classification science, this instability is expected to continue in the classification of scorpions, and in the future, the movement of species among the families will occur. One of the main reasons for the instability of the classification of these arthropods is the changes in faunistic species structure in different climatic and geographic regions of Iran. Solving this problem involves conducting studies using new species identification methods, such as molecular and genetic methods that can solve the classification of scorpions.

According to the latest studies, the checklist of scorpions in Iran consists of three families: Buthidae, Scorpionidae, and Hemiscorpionidae with 20 genera and 64 species. The last change occurred in the genus Nebo, an unknown new species was reported in Jiroft County, Kerman province (98). At present, the number of species of the genus *Odontobuthus* has reached five species (75, 77).

Several species of the scorpions have a toxic and fatal sting and others have a painful sting. These species are more likely to be in contact with humans and their dwellings; that is why they are classified as medically important scorpions. In Iran, 12 species of eight genera are as follows: *Hemiscorpius, Androctonus*, *Odontobuthus*, *Apistobuthus*, *Compsobuthus*, *Hottentotta*, *Orthochirus*, and *Mesobuthus* (99).

Blood hypotension, accelerated heart rate, seizure, anesthesia, distraction, agitation and anxiety, hemolysis, ulcers and skin necrosis, and renal failure have been reported with medically important scorpion sting (100). In the scorpion breeding places in Iran, especially in the hot areas of the Khuzestan and Isfahan provinces in central and southwest of the country, there are a number of animals that feed on scorpions and are considered their predators (101, 102). The scorpion sting of the family Buthidae is painful (103). After being stung by these scorpions, swelling and numbness are observed at the stung area and the pain that feels most at night. Some species of this family are noticeable because of their medical importance.

One of the most harmful and fatal species of this family in Iran is *Androctonus crassicauda*. Mortality reports of this species have been reported in the West Azarbaijan, East Azarbaijan, and Khuzestan provinces (60, 95, 96). The *Orthochirus scrobiculosus* is another toxic scorpion of familiy Buthidae, which is one of the most medically important scorpions in Iran. A mortality report from this species was recorded in Khuzestan province in southwest of Iran (5). The *Hemiscorpius lepturus* of family Hemiscorpiidae has high medical importance and is the most dangerous scorpions in Iran, which annually causes a number of mortalities among children and adults in the southern (Hormozgan province) and southwest (Khuzestan province) regions of Iran (96, 97). Ultimately, there is a mortality report of the *H. acanthocercus* of family Hemiscorpiidae in southern Iran (Hormozgan province) (95). The family Scorpionidae in Iran with having three species has little medical importance. Of family Scorpionidae, *Nebo henjamicus* was reported for the first time by Francke (1980), from the Hengam Island in the Persian Gulf (98). After that, few samples of this species have been reported from the Iranian plateau in Kerman Province, south east of Iran, which needs to be investigated further (98). The southern and southwestern regions of the country are rich concerning scorpion species (26, 34, 94, 104). Scorpions, such as other arthropods and animals, are the major health pests that cause discomfort among people in different areas of the country (105, 20).

Therefore, applying the control methods, including the use of chemical pesticides, are also considered, although the issue of resistance and environmental pollution is a major problem in the use of pesticides, which applies to all arthropods, such as scorpions. The use of pesticides to control these creatures should be carefully and accurately planned (106, 107).

## Conclusion

The highest diversity and abundance of scorpion species are in southern and southwest regions in Iran, especially Hormozgan and Khuzestan provinces. However, the diversity of scorpions is less, from the southwest to the north and northwest of the country. However, the completion of information on scorpion species in Iran requires more effort by the researchers.

## Ethical considerations

Ethical issues (including plagiarism, informed consent, misconduct, data fabrication and/or falsification, double publication and/or submission, redundancy, etc.) have been completely observed by the authors.
